# COVID-19 Vaccines in Children

**DOI:** 10.3390/jcm13010087

**Published:** 2023-12-23

**Authors:** Danielle Fayad, Robert W. Frenck

**Affiliations:** Division of Infectious Diseases, Department of Pediatrics, Cincinnati Children’s Hospital, University of Cincinnati, Cincinnati, OH 45221, USA; danielle.fayad@cchmc.org

**Keywords:** COVID-19, pediatric COVID-19, COVID-19 in children, COVID-19 vaccines

## Abstract

The COVID-19 pandemic has left an indelible mark on global health, affecting individuals of all ages across diverse communities. While the virus has predominantly been associated with severe outcomes in adults, its impact on children has garnered increasing attention. Today, three COVID-19 vaccines are available for use in the U.S. and recommended by the Advisory Committee on Immunization Practices (ACIP). As of September 2023, ongoing genomic surveillance identified SARS-CoV-2 XBB sublineages as the most common circulating SARS-CoV-2 variants, constituting over 99% of sequenced SARS-CoV-2 specimens in the US. Recently, recommendations for COVID-19 vaccination were updated accordingly to the 2023–2024 Omicron-XBB.1.5-adapted monovalent COVID-19 vaccine to provide heightened protection against currently circulating SARS-CoV-2 XBB-sublineage variants. COVID-19 vaccines have proven to be safe, efficacious, and effective at protecting against COVID-19 and preventing severe illness in children and adolescents.

## 1. Introduction

As of September 2023, more than 17 million cases of severe acute respiratory syndrome coronavirus 2 (SARS-CoV-2) infection have been documented in children aged <18 years in the U.S., resulting in at least 30,000 COVID-associated hospitalizations and 2000 COVID-19-related deaths [1]. To combat this pandemic, efforts were expedited to create safe and efficient vaccines against SARS-CoV-2, and by late 2020, numerous vaccine candidates for use in adults obtained emergency use authorization worldwide. The pediatric vaccine scene was slower to progress [2]. While COVID-19 hospitalization rates are lower in children compared to adults, they still approach the rates seen in the past for diseases that are now preventable through vaccination [1,2,3,4,5]. Today, three COVID-19 vaccines are available for use in the U.S. and recommended by the Advisory Committee on Immunization Practices (ACIP). These include the updated (2023–2024) monovalent mRNA Pfizer-BioNTech COVID-19 vaccine, the updated monovalent mRNA Moderna COVID-19 vaccine, and the protein-subunit-based Novavax COVID-19 vaccine [6]. To date, few studies have delved into the comprehensive literature on COVID-19 vaccination in children. In this review, we will explore the safety, efficacy, and effectiveness of COVID-19 vaccines authorized for use in children aged 6 months to 18 years in the U.S.

## 2. Epidemiology of Pediatric COVID-19 Illness

COVID-19 has the potential to cause severe disease in children [1,3,7]. Although children with underlying medical conditions are at increased risk for serious disease, healthy children can also experience severe illness and complications including acute respiratory distress syndrome, myocarditis, organ failure, and multisystem inflammatory syndrome in children (MIS-C) [8,9,10]. Among children hospitalized with COVID-19 to date, up to 32% required care in an intensive care unit, 4% to 14% required invasive mechanical ventilation, and up to 2000 children have died [1]. As Table 1 shows, various vaccine-preventable diseases, for which vaccination is currently recommended, had comparable and even lower annual pediatric hospitalization and mortality rates prior to vaccine licensure and widespread implementation. Additionally, the incidence of COVID-19 among young children is notably higher when compared to other childhood illnesses, which can contribute to its rapid spread to a large population [1]. Rates of COVID-19-related hospitalizations in children and adolescents peaked during the Delta- and Omicron-predominant periods (ending September 2021 and beginning January 2022, respectively), and higher rates were documented in unvaccinated adolescents compared to fully vaccinated adolescents [5]. With the increased epidemiological burden of COVID-19, children and adolescents have also been impacted by the long-term health effects of this virus. Up to 16% of children and adolescents with COVID-19 continue to experience persistent symptoms beyond 3 months post infection [11].

As part of the national public health emergency declared in response to the COVID-19 pandemic, the Centers for Disease Control and Prevention (CDC) under the guidance of the ACIP has provided evidence-based recommendations regarding the utilization of COVID-19 vaccines within the U.S. following each regulatory decision made by the Food and Drug Administration (FDA). Today, three COVID-19 vaccines are available for use in the U.S. and recommended by the ACIP. These include the updated (2023–2024) monovalent mRNA Pfizer-BioNTech COVID-19 vaccine, the updated monovalent mRNA Moderna COVID-19 vaccine, and the protein-subunit-based Novavax COVID-19 vaccine [6]. As of September 2023, bivalent mRNA COVID-19 vaccines are no longer recommended in the U.S. [6,21]. Janssen (Johnson and Johnson, New Brunswick, NJ, USA) adenovirus viral vector COVID-19 vaccine is no longer available in the U.S., and remaining government stock expired in May 2023 [22]. Other types of vaccines have been developed and are used in other regions of the world. As of September 2023, a total of 183 SARS-CoV2 vaccines are in clinical development [23].

## 3. Vaccine Types

Types of COVID-19 vaccines available in the U.S. are included in Table 2.

### 3.1. mRNA COVID-19 Vaccines (Moderna, Pfizer-BioNTech)

BNT162b2 (Pfizer-BioNTech, Mainz, Germany) and mRNA-1273 (Moderna, Cambridge, MA, USA) vaccines are lipid nanoparticle formulations containing nucleoside-modified mRNA encoding the SARS-CoV-2 viral full-length spike glycoprotein [26].

In June 2022, the original monovalent mRNA COVID vaccines (containing components from the ancestral strain of SARS-CoV-2) were approved as primary series for children aged 6 months–4 years (Pfizer/BioNTech, BNT162b2) and 6 months–5 years (Moderna, mRNA-1273) [1]. In the following months, the FDA amended its emergency use authorization (EUAs) to authorize the use of age-appropriate, updated bivalent mRNA vaccines (containing components from the ancestral and Omicron BA.4 and BA.5 strains in equal amounts). The FDA subsequently expanded authorization for use of bivalent vaccines to children 6 months of age and older, with a corresponding recommendation for use from the ACIP [27,28].

On 12 September 2023, the ACIP provided a universal recommendation for the use of the updated 2023–2024 Omicron-XBB.1.5-adapted monovalent COVID-19 vaccines in people 6 months of age and older [6,21].

### 3.2. Protein Sub-Unit Vaccine (Novavax)

NVX-CoV2373 (Novavax, Gaithersburg, MD, USA) is a recombinant spike protein nanoparticle vaccine with matrix-M adjuvant [25].

In August 2022, the FDA provided emergency use authorization (EUA) for the use of Novavax as a two-dose monovalent vaccination series in individuals 12 through 17 years of age and as a monovalent booster in adults who do not want to receive an mRNA bivalent vaccine [25]. The updated version of the Novavax vaccine targeting the XBB strain is currently under review by the FDA for EUA. To date, the Novavax COVID-19 vaccines have not received licensure from the FDA [25].

### 3.3. Live Viral Vector Vaccine (Janssen/Johnson & Johnson)

A recombinant, replication-incompetent, human adenovirus type 26 vector encoding the SARS-CoV-2 spike protein, the Ad26.COV2.S COVID-19 vaccine (from Johnson & Johnson and Janssen, Beerse, Belgium) was the only viral vectored vaccine that became available in the U.S. under EUA [29,30].

The Janssen COVID-19 vaccine received EUA status in February 2021. However, after the vaccine was available for 2 months, safety signals of thrombosis with thrombocytopenia syndrome (TTS) arose, leading the FDA to pause use of the vaccine while the signals were being investigated [31]. Initial findings led to the pause being lifted 10 days later [31]. However, as the vaccine was used again, additional cases of TTS were identified, which led to limitation of the EUA authorization and eventually to the FDA rescinding the EUA for this vaccine in June 2023 [29]. Although initial testing of the Janssen vaccine in children was initiated, the vaccine was never approved by the FDA for use in individuals younger than 18 years of age [29].

## 4. Safety Data

Safety data is obtained through a combination of pre-authorization clinical trials and post-vaccine authorization and licensure surveillance data. The former is to identify more common adverse events (AEs), while the latter focus on identifying potential rare AEs not identified during clinical trials of the vaccine. The safety data we will explore are available through the Vaccine Adverse Event Reporting System (VAERS), the Vaccine Safety Link (VSD), and the Clinical Immunization Safety Assessment (CISA) Project [32]. In contrast to VAERS, which relies on passive reporting, VSD cases are identified through active surveillance. For COVID-19 vaccine safety surveillance, the CDC also established an additional system called v-safe that uses smartphone-based technology to capture safety data [32].

### 4.1. Safety in Clinical Trials

#### 4.1.1. NVX-CoV2373 (Novavax)

As part of the phase 3, randomized, observer-blinded, placebo-controlled multicenter clinical trial termed PREVENT-19, the NVX-CoV2373 vaccine was evaluated in adolescents between the ages of 12 and 17 years [33]. As demonstrated in Table 3, local and systemic events were generally mild to moderate and transient [33]. Serious AEs were rare, and no episodes of anaphylaxis, Guillain Barré syndrome (GBS), TTS, or myocarditis/pericarditis were observed (Table 3) [33].

#### 4.1.2. mRNA-1273 Vaccine (Moderna)

- Children 12 years to 17 years

The initial evaluation of the Moderna mRNA-1273 vaccine was in TeenCOVE, an observer-blinded, placebo-controlled trial evaluating the safety, immunogenicity, and efficacy of the vaccine in adolescents 12 to 17 years of age [34]. A total of 3732 participants were randomly assigned to receive either two 50 μg doses of mRNA-1273 vaccine (2489 participants) or placebo (1243 participants) [34]. The most common solicitated AEs were injection-site pain (in >90% after first of second injection), headache, and fatigue (Table 3) [34]. Most local and systemic AE were grade 1, and no cases of myocarditis or pericarditis were reported [34].

- Children 6 years to 11 years

Based on the excellent safety of the vaccine in adolescents, evaluation of mRNA-1273 was age de-escalated to children 6–11 years of age (up to 12th birthday at time of enrollment) in the KidCOVE study. In a 3:1 manner, 4016 children were randomized to vaccine or placebo to evaluate the safety, immunogenicity, and efficacy of the mRNA-1273 vaccine. Children received either two 50 μg injections of mRNA-1273 or placebo [35]. The most common AE was injection-site pain, and reactogenic events were mostly mild to moderate [35]. As compared to vaccine recipients 16–25 years of age, the incidence of grade 3 AEs was lower in children 6–11 years of age (Table 3) [34,36]. No deaths, anaphylaxis events, MIS-C, myocarditis, pericarditis, or vaccine-related serious AEs were reported [35].

- Children 6 months to 5 years

Following availability of the initial safety reports of children 5–11 years of age who received mRNA-1273, the KidCOVE trial was further age de-escalated to include children 6 months to 5 years of age (up to 6th birthday at the time of enrollment). In the expanded trial, 3040 children 2 years to 5 years of age and 1762 children 6 months to 23 months of age were randomly assigned to receive two 25 μg injections of mRNA-1273 (half the dose administered to people 6 years of age and above) or placebo [37]. The reactogenicity in children 6 months to 5 years was lower than that observed in children 6 years of age and above (Table 3) [34,35,37]. The most common systemic AEs among children 2 years to 5 years of age was fatigue, while in children 6 months to 2 years, the most common reactions were irritability, sleepiness, and loss of appetite [37]. In both the 6-to-23-month and 2-to-5-year cohorts, the incidence of fever among vaccine recipients was around 20%, similar to that after other routinely recommended vaccines. No cases of myocarditis, pericarditis, or MIS-C and no deaths were reported [37].

#### 4.1.3. BNT162b2 Vaccine (Pfizer/BioNTech)

- Children 16 years to 17 years

Based on the safety and immunogenicity of the 30 μg dosage of BNT162b2 in adults, along with the supposition that 16-year-olds physiologically and morphologically are very similar to adults, the initial COVID-19 vaccine trial in adults was expanded to adolescents at least 16 years of age [38]. In this double-blind, placebo-controlled study, the adverse event profile in adolescents closely mirrored the profile in adults, with transient mild-to-moderate symptoms such as pain at the injection site, fatigue, and headache being the most commonly reported symptoms following vaccination (Table 3) [38]. During the clinical trial, serious AEs among vaccine recipients were infrequent and statistically equivalent to the placebo group [38].

- Children 12 years to 15 years

Similar to the mRNA-1273 trials, BNT162b2 was evaluated in children in an age de-escalating manner. When safety and preliminary immunogenicity data were available in the older teens, 2260 adolescents 12 to 15 years of age were randomly assigned to receive the 30 μg dosage of BNT162b2 or placebo [39]. Similar to adults and older teens, local and systemic events were generally mild to moderate and lasted less than 3 days (Table 3) [39]. Few participants (<0.4%) had serious AEs, none of which were deemed to be vaccine related [39].

- Children 5 years to 11 years

Due to the potential for a significant difference in size in children under 12 as compared to adults when vaccination was expanded to children 5 years to 11 years of age, a dose-ranging study was initially conducted that determined a 10 ug dosage was optimal for this age range [40]. Following selection of the 10 ug dosage, 2268 children were randomly assigned to receive the BNT162b2 vaccine or placebo [40]. As in older vaccinees, in children 5 years to 11 years of age, local and systemic events were generally mild to moderate and lasted less than 3 days (Table 3) [40]. In comparison to adults and adolescents, children 5 to 11 years old generally experienced fewer systemic events such as fever and chills but had a higher occurrence of injection-site redness and swelling [38,39]. The design of the study precluded the ability to determine if the lower incidence of systemic events was related to the age of the children or that they received one-third the dose of adolescents and adults. Three serious AEs were reported, none of which were considered to be related to vaccine or placebo [40]. No myocarditis, pericarditis, MIS-C, or anaphylaxis in BNT162b2 recipients was reported [40].

- Children 6 months to 4 years

Additional dose-ranging studies were conducted for children under 5 years of age, and three doses of a 3 ug dosage of BNT162b2 was selected for use in the larger trial [41]. Initially, children 2 years to 4 years of age (up to the 5th birthday at the time of enrollment) were randomized 2:1 to receive vaccine or placebo (1835 vaccinees:915 placebo). Subsequently, 1178 children 6 months to less than 2 years of age were administered vaccine, and 598 were administered placebo [41]. Again, reported AEs to BNT162b2 were generally mild to moderate, and no grade 4 events were reported (Table 3) [41]. Active surveillance was conducted for myocarditis, pericarditis, facial paralysis, thromboembolic events, thrombocytopenic events, MIS-C, and vaccine-related anaphylaxis. While the sample size was relatively small, none of the previously listed outcomes were recorded [41].

### 4.2. Safety in Post-EUA Surveillance

#### 4.2.1. NVX-CoV2373 (Novavax)

In the U.S., between July 2022 and March 2023, 69,227 people 12 years of age and above were administered a dose of NVX-CoV2373. Of those, 230 reports of adverse events (AEs) were made to VAERS, 201 (91.7%) of which were classified as non-serious (not requiring hospitalization or resulting in life-threatening illness or death) [42]. The most commonly reported AEs included dizziness (14.3%), fatigue (11.3%), and headache (10.9%) [42]. Nineteen (8.3%) AEs were classified as serious and included one case of thrombosis, two of pericarditis, one of Guillain–Barré syndrome, and two of seizure. On review of medical records, thrombosis and pericarditis cases were determined to be due to another potential underlying cause [42]. No deaths related to NVX-CoV2373 were reported.

#### 4.2.2. mRNA Vaccines

- Children 12 years to 17 years

In an early interim analysis of VSD surveillance data, Klein et al. monitored the incidence of 23 events including bell palsy, Guillain–Barré syndrome (GBS), and myocarditis/pericarditis among mRNA vaccine recipients [43]. Following administration of 626,456 mRNA vaccine doses in children 12–17 years of age, no significant associations between vaccination with mRNA COVID-19 vaccines were detected [43]. In another report of VAERS surveillance data following administration of 1464 BNT162b2 bivalent booster doses to 12- to 17-year-olds, most AEs reported were non-serious and included headache (11.9%), fatigue (10.9%), and fever (10.6%) [44]. Given concern for the higher-than-expected number of myocarditis reports following mRNA vaccination in adults, Goddard et al. studied the incidence of myocarditis/pericarditis specifically and reported a higher incidence after first boosters than after primary series doses [45]. In all age groups, the incidence of myocarditis was higher in males than in females, with the highest incidence (188 cases per million doses) occurring in adolescent males 16 to 17 years old [45]. Review of VAERS reports identified a similar age and sex distribution of myocarditis/pericarditis but found a lower incidence rates than in the Goddard study (71 and 106 cases per million doses of the BNT162b2 vaccine, in 12-to-15- and 16-to-17-year-olds, respectively) [46]. VAERS surveillance data from December 2020 to August 2021 found a similar incidence of myocarditis/pericarditis as previous reports [47]. However, risk of myocarditis/pericarditis associated with BNT162b2 vaccine was higher as compared to mRNA-1273 vaccination (reporting odds ratios of 5.37 following BNT162b2 and 2.91 following mRNA-1273) [47].

An important factor is to compare the rates of myocarditis following vaccination to the background rate. In a retrospective analysis of 60,390,000 children hospitalized between 2007 and 2016, Vasudeva et al. estimated the incidence of acute myocarditis to be 150 per million in children aged 15–18 years, with the highest incidence of 180 per million recorded in 2015–2016 [48], closely mirroring the incidence of myocarditis following mRNA vaccination [45,46,48]. Moreover, compared to cases of vaccination-related myocarditis, myocarditis associated with infections typically have a more indolent presentation and complicated recovery [46,49]. Among 514 children hospitalized with acute non-vaccine-related myocarditis between 2006 and 2011, 44.4% required mechanical ventilation, 18.9% received extracorporeal membrane oxygenation (ECMO), 4.1% received a heart transplant, and 7.2% died. In contrast, among 762 patients hospitalized with myocarditis following mRNA vaccination, 0.3% required mechanical ventilation, none required ECMO or heart transplantation, and no deaths occurred [46].

- Children 5 years to 11 years

In January 2023, preliminary findings from v-safe and VAERS for bivalent mRNA booster vaccination in children were reported [50]. Following administration of bivalent BNT162b2 vaccine to 861,251 children (5–11 years) and bivalent mRNA-1273 vaccine to 92,108 children (6–11 years), AEs were similar to those noted during the TEENCove and KIDCove trials, with the most common event being injection-site reactions [50]. No reports of myocarditis were received [50]. To better understand the incidence of myocarditis/pericarditis following COVID-19 vaccination, a VSD study was performed evaluating cases among 5- to 39-year-olds presenting to emergency departments (ED) and inpatient settings within 7 days of vaccination [45]. Following the administration of 948,191 doses of BNT162b2, three cases of myocarditis were reported, suggesting an incidence of 3.1 per million doses, mirroring the background incidence of myocarditis in this age group [45,48].

- Children 6 months to 5 years

Among 135,005 children 6 months to 4 years of age who received BNT162b2 and 112,006 children 6 months to 5 years of age who received mRNA-1273, no cases of vaccine-related myocarditis or pericarditis were detected [51]. Single cases of hemorrhagic stroke, pulmonary embolism, and MIS-C were identified; however, none were deemed related to vaccination [51]. Aligning with the findings from v-safe and VAERS, systemic reactions were more commonly reported in children aged 6 months to 2 years than for children aged 3 to 5 years [36]. However, over 98% of reactions were nonserious [36]. After administration of approximately 550,000 third doses of mRNA COVID-19 vaccine to children aged 6 months–5 years, eight serious AEs were received by VAERS and included a case of acute hemorrhagic edema of infancy, diabetic ketoacidosis, Henoch–Schönlein purpura, Kawasaki disease, new onset afebrile seizure (two cases), pneumonia, and asthma exacerbation [52]. After reviewing available clinical information, no indications were found to suggest that these reported events were connected to vaccination [52]. No cases of myocarditis or pericarditis were identified [52].

## 5. Efficacy Data

### 5.1. NVX-CoV2373 (Novavax)

In the PREVENT-19 trial, 20 mild COVID-19 cases occurred, 14 of which were among placebo recipients [33]. A protective vaccine efficacy of 79.5% (95% CI, 46.8–92.1) was demonstrated in adolescents [33]. This finding aligns with that observed in the adult portion of this trial [33,53] as well for other mRNA vaccines in this age group [34,39]. Vaccine efficacy for the Delta variant was 82.0% (95% CI, 32.4–95.2) [33].

### 5.2. mRNA-1273 Vaccine (Moderna)

- Children 12 years to 17 years

In the TeenCOVE trial, vaccine efficacy of mRNA-1273 against COVID-19 with an onset of 14 days after the first dose was 92.7% (95% CI, 67.8–99.2) (Table 4) [34]. Vaccine efficacy of mRNA-1273 for asymptomatic infection with an onset 14 days after the second dose was 59.5% (95% CI, 28.4–77.3) [34].

- Children 6 years to 11 years

In 6- to 11-year-olds, the estimated vaccine efficacy against COVID-19 occurring 14 days or more after the first dose was 88.0% (95% CI, 70.0–95.8) (Table 4) [35]. This finding was during a period when the dominant circulating variant was B.1.617.2 (Delta) variant [35].

- Children 6 months to 5 years

mRNA-1273 vaccine efficacy was evaluated in the KidCOVE trial in this age group, at a time when B.1.1.529 (Omicron) was the predominant circulating variant [37]. The estimated vaccine efficacy against COVID-19 for 2- to 5-year-olds was 36.8% (95% CI, 12.5–54.0), while for 6-to-23-month-olds, it was 50.6% (95% CI, 21.4–68.6) [37]. The estimated vaccine efficacy was lower in this study and age group compared to earlier trials in children older than 5 years of age conducted at a time when other variants were prevalent [34,35]. It is likely that the lower efficacy was due to the circulating variant at the time of the clinical trial and can be attributed to the fact that the initial vaccine administered was based on the original COVID-19 strain. Despite the substantial mutations in the virus, the original vaccine demonstrated reasonable protection against infection.

### 5.3. BNT162b2 Vaccine (Pfizer/BioNTech)

- Children 16 years to 17 years

Efficacy in this age group was evaluated as part of a larger multinational trial including persons 16 years of age or older [38]. Observed vaccine efficacy in 16-to-65-year-olds was 95.6% (95% CI, 89.4–98.6) (Table 4). Through 6 months of follow up, vaccine efficacy against COVID-19 was 91.3% (95% CI, 89.0 to 93.2) among participants without evidence of previous infection [54]. Note that this study was conducted prior to the existence of the Delta or Omicron variants of SARS-CoV-2.

- Children 12 years to 15 years

Defined as onset of symptoms beginning 7 or more days after the second dose of vaccine, no case of COVID-19 was recorded among the 1131 children who received BNT162b2, as compared to 16 cases that occurred among the 1129 children who received placebo [39]. While the absolute vaccine efficacy was 100%, the confidence intervals were wide (95% CI, 75.3–100) [39]. Thus, a more practical interpretation of the data would be that the vaccine likely was at least as effective as in older teens and adults.

- Children 5 years to 11 years

Using the same definition as outlined for 12–15-year-olds, three children 5 years to 11 years of age who received BNT162b2 developed COVID-19, as compared to 16 cases among the placebo recipients, resulting in an observed vaccine efficacy of 90.7% (95% CI, 67.7–98.3) (Table 4) [51].

- Children 6 months to 4 years

By the time the clinical trials had advanced to including children 6 months to 4 years of age, the Delta and then Omicron variants were common [41]. Among the children receiving BNT162b2, there were 13 cases of COVID-19, as compared to 21 cases in the placebo group, with a resultant vaccine efficacy of 73.2% (95% CI, 43.8 -87.6) [41]. Further breakdown by age showed an observed vaccine efficacy of 75.8% (95% CI, 9.7–94.7) for children aged 6 months to less than 2 years, and 71.8% (95% CI, 28.6–89.4) for children aged 2 to 4 years (Table 4) [41]. While efficacy in this age group was lower than in previous groups, this is most likely due to Omicron being the predominant variant during the trial. The relatively high efficacy against Omicron is very encouraging for a vaccine targeted against the ancestral stain of the virus.

## 6. COVID-19 Effectiveness Data

Compared to efficacy data collected during clinical trials, effectiveness data reflect the “real-world” protection provided by a vaccine. Numerous effectiveness studies have been conducted among adolescents 12 to 17 years of age [55,56,57].

During pre-Delta-, Delta-, and Omicron-predominant periods, BNT162b2 VE estimates against COVID-19-associated emergency department or urgent care (ED/UC) encounters for the two-dose regimen were higher (73–93%) shortly after the second dose and significantly declined at more than 150 days after vaccination (13–72%) [56,57,58]. A third (booster) dose of vaccine increased VE among 12-to-15-year-olds and adolescents aged 16–17 years to 54% and 86%, respectively [57,58]. Comparing vaccine effectiveness over time, Fowlkes et al. demonstrated that VE was more related to the variant circulating than the time post-vaccination [55]. While VE against Delta was 87% over the period of 14–149 days after vaccination, VE against Omicron infection over a similar time period was only 59% [55]. However, if the period after vaccination exceeded 150 days, adjusted VE against Delta and Omicron variants were very similar (VE of 60% against Delta vs. 62% against Omicron) [55]. Receipt of two BNT162b2 vaccine doses in adolescents 12 years of age and above provided significant protection against COVID-19-associated hospitalizations (>90%), ICU admission (98%), requiring life support (>90%), and MIS-C (>90%) [57,58,59,60].

Much less effectiveness data are available for children under 12 years of age. VE against COVID-19-associated ED/UC encounters was evaluated as part of the VISION network [58]. Among children aged 5 to 11 years, two doses of BNT162b2 resulted in a VE against COVID-19 ED/UC of 46–49% [58]. It is important to note that the study was performed during an Omicron-predominant period [58]. VE decreased slightly at more than 150 days post vaccination to 41% [58]. A similar VE of 31% against symptomatic and asymptomatic Omicron infection in children who received two doses of BNT162b2 was reported in the PROTECT study, a prospective cohort of 1364 children and adolescents aged 5 to 15 years [55]. Through the VISION network, VE against COVID-19-associated ED/UC encounters during the full study period, which included pre-Delta-, Delta-, and Omicron-predominant periods, was 74% after the second dose of BNT162b2 among children aged 5 to 11 years [57].

In a multi-site analysis of medically attended visits among children aged 6 months to 5 years as part of the VISION network, VE against ED/UC encounters was 29% at ≥14 days after second monovalent mRNA-1273 vaccine [61]. In children aged 6 months to 4 years, VE against ED/UC encounters was 43% at ≥14 days after a third monovalent BNT162b2 dose [61]. Data from the New Vaccine Surveillance Network (NVSN; an active, prospective, population-based surveillance network for pediatric viral infections at seven US medical centers) reported comparable VE of 30–40% against ED visits and hospitalization following > 1 COVID-19 vaccine of any product in children aged 6 months to 4 years (ACIP September 2023). When the incidence of COVID-19 infection among 318 children 6 months to 5 years of age who received ≥ 1 bivalent vaccine dose was compared to 30,146 unvaccinated children of the same age, regardless of the vaccine product, there was an 80% decrease in infection among vaccinated children [61]. A limitation of the study was the relatively small sample size.

## 7. Limitations

The safety data presented in this study, including VAERS and CISA, draw extensively from adverse events reported to passive surveillance systems. While these systems play a crucial role in monitoring vaccine safety, it is imperative to acknowledge their inherent limitations and biases [32]. One of the primary limitations of passive reporting systems is the uncertainty of the accuracy of reporting. These systems are susceptible to both over- and under-reporting [32]. The inherent uncertainties of reporting along with a lack of denominator data provides challenges in calculating true incidence rates [32]. In light of these limitations, it is crucial to interpret the safety data cautiously. While passive reporting systems offer valuable insights, they should be complemented with other methodologies, such as active surveillance and epidemiological studies, to provide a more comprehensive understanding of vaccine safety.

## 8. Future Projections

The SARS-CoV-2 Omicron XBB.1.5 variant is a sublineage of XBB, which is a recombinant of two BA.2 sublineages [1]. It was first detected in the U.S. in October 2022, and by early January 2023, up to 5000 sequences of this variant had been reported from 38 countries, with most originating in the US [62]. By the end of January 2023, XBB.1.5 became the predominant circulating variant in the U.S. As of September 2023, SARS-CoV-2 XBB-sublineage variants accounted for >99% of sequenced SARS-CoV-2 specimens in the U.S. [63]. While Omicron BA.4/BA.5-adapted bivalent COVID-19 vaccines do provide a degree of protection against various outcomes related to XBB-related COVID-19, recent evidence indicates that vaccines that are better tailored to the strains currently in circulation can offer enhanced protection against symptomatic and severe disease [64]. On 12 September 2023, the CDC recommended the Omicron-XBB.1.5-adapted monovalent COVID-19 updated for 2023–2024 for everyone aged 6 months and older in an attempt to enhance vaccine-induced immunity and provide heightened protection (compared with waning protection from earlier vaccines) against currently circulating SARS-CoV-2 XBB-sublineage variants [6].

## 9. Conclusions

COVID-19 has the potential to cause severe disease in children. Demonstrating both safety and efficacy, vaccines against COVID-19 stand as a robust defense against the infection. Ongoing genomic surveillance has identified the emergence of SARS-CoV-2 XBB sublineage variants in 2023. Recommendations for COVID-19 vaccination were updated accordingly to the 2023–2024 Omicron-XBB.1.5-adapted monovalent COVID-19 vaccine to provide heightened protection against currently circulating SARS-CoV-2 XBB-sublineage variants. As we navigate the dynamic and ever-changing landscape of the pandemic, it is paramount to underscore the importance of continued monitoring and active research to best serve our communities.

## Figures and Tables

**Table 1:** Incidence rate, hospitalizations, and deaths from COVID-19 in comparison to other childhood diseases pre-vaccine licensure.

Virus	Yearly Incidence Rate/Year, <18 Year	Hospitalizations/Year	Deaths
COVID-19	100–1900 per 100,000; 2020–2023 [1]	9–34 per 100,000, 0–17 year, 2020–2023 [1]	2320 children, 2020–2023 [1]
Varicella	Not available	4–31 per 100,000; <20 year, 1988–1999 [12]	50 children per year, 1970–1994 [13]
Influenza	20–30 per 100,000; 2010–2021 [14]	20 per 100,000, 0–17 year, 2017–2018 [15]	171 children, 2017–2018 [16]
Rubella	~0.5–1.5 per 100,000; prior to vaccine licensure in 1969 [17]	Not available	17 children per year, 1966–1968 [18]
Hepatitis A	~0.2–0.6 per 100,000; prior to vaccine licensure in 1995 [17]	<1 per 100,000; 5–14 year, 2005 [19]	3 children per year, 1990–1995 [20]

**Table 2:** Characteristics and simplified schedule of major COVID-19 vaccines.

Vaccine	BNT162B2	MRNA-1273	NVX-COV2373
Manufacturer	Pfizer/BioNTech	Moderna	Novavax
Type of Vaccine	mRNA vaccine	mRNA vaccine	Recombinant subunit adjuvanted protein vaccine
Antigen	Full-length spike protein Monovalent, Omicron XBB.1.5	Full-length spike protein Monovalent, Omicron XBB.1.5	Full-length spike glycoprotein of prototype strain plus Matrix-M adjuvant Monovalent
Recommended Doses in Patients Without Moderate or Severe Immunocompromise	6 m–4 y: 3-dose series (1 or 2 doses if previously vaccinated with >1 or 1 dose of any BNT162b2 vaccine except updated 2023–2024 vaccine, respectively) ≥5 y: 1-dose	6 m–5 y: 2-dose series (1 dose if previously vaccinated with ≥ 1 dose of any mRNA-1273 vaccine except updated 2023–24 vaccine) ≥6 y: 1-dose	≥12 y: 2-dose series separated by 3–8 weeks
Recommended DOSES in Patients with Moderate or Severe Immunocompromise	6 m–4 y: 3-dose series (1 or 2 doses if previously vaccinated with >1 or 1 dose of any BNT162b2 vaccine except updated 2023–2024 vaccine, respectively) * ≥5 y: 3-dose series (1 or 2 doses if previously vaccinated with >1 or 1 dose of any BNT162b2 vaccine except updated 2023–2024 vaccine, respectively) *	≥6 m: 3-dose series (1 or 2 doses if previously vaccinated with >1 or 1 dose of any mRNA-1273 vaccine except updated 2023–2024 vaccine, respectively) *	≥12 y: 2-dose series separated by 3–8 weeks
Other Vaccines Using Technology	None	None	None
References	[24]	[24]	[24,25]

* Additional dose of monovalent mRNA vaccine is an option, informed by provider clinical judgement and at least 8 weeks after last dose.

**Table 3:** Local and systemic adverse events (AEs) reported within 7 days after administration of vaccine dose, by vaccine type, and age group.

Vaccine Type, Manufacturer		mRNA-1273, Moderna				BNT162b2, Pfizer/BioNTech					**NVX-CoV2373, Novavax**
	Age Group	6 Month–23 Month	2 Year–5 Year	6 Year–11 Year	12 Year–17 Year	6 Month–23 Month	2 Year–4 Year	5 Year–11 Year	**12 Year–15 Year**	**16 Year–25 Year**	**12 Year–17 Year**
AEs											
Local AEs											
Any symptom		44–54	57–75	94–95	93–94	NA	NA	NA	NA	NA	65–75
Injection-site pain		37–46	53–68	93–95	92–93	15–17	27–31	71–74	79–86	78–83	56–65
Erythema		9–14	4–12	12–19	13–19	7–11	9–11	15–19	5–6	6	1–7.5
Swelling		8–15	3–12	12–17	16–20	3–4	3–6	10–15	5–7	7–8	1–8
Lymphadenopathy		NA	NA	16–18	21–23	NA	NA	NA	NA	NA	NA
Systemic AEs											
Any symptoms		44–54	57–75	58–78	68–86	NA	NA	NA	NA	NA	55–74
Fever		11–15	8–19	3–24	2–12	6–7	4–5	3–7	10–20	7–17	1–17
Irritability/crying		64–67	54–55 ^a^	NA	NA	44–51	NA	NA	NA	NA	NA
Sleepiness/drowsiness		35–37	30–36 ^a^	NA	NA	20–27	NA	NA	NA	NA	NA
Decreased appetite		30–32	24–31 ^a^	NA	NA	20–22	NA	NA	NA	NA	NA
Headache		NA	12–16 ^b^	31–54	45–70	NA	4–5	22–28	55–65	54–61	30–57
Fatigue		NA	40–48 ^b^	43–65	48–68	NA	24–30	34–39	60–66	60–66	24–50
Myalgia		NA	10–16 ^b^	15–28	27–47	NA	2–3	9–12	24–32	27–41	34–59
Arthralgia		NA	6–9 ^b^	9–16	15–29	NA	1–2	3–5	10–16	13–22	7–16
Nausea/Vomiting		NA	7–10 ^b^	11–24	11–24	NA	2–3	1–2	2–3	2–3	8–20
Diarrhea		NA	NA	NA	NA	NA	5–8	5–6	6–8	8–11	NA
Chills		NA	6–12 ^b^	10–31	18–43	NA	2–3	5–10	28–42	25–40	NA

^a^ Data not available in children 37 months to 5 years of age; ^b^ data only available in children 37 months to 5 years of age; NA, data not available for review.

**Table 4:** Vaccine efficacy, by vaccine type and age group.

	Age Groups			
Vaccine Efficacy (95% CI) by Vaccine Type	6 Month–23 Month	2 Year–5 Year (mRNA-1273) 2 Year–4 Year (BNT162b2)	6 Year–11 Year (mRNA-1273) 5 Year–11 Year (BNT162b2)	12 Year–17 Year (mRNA-1273) >12 Year (BNT162b2, NVX-CoV2373)
mRNA-1273, Moderna	50.6% (21.4–68.6)	36.8% (12.5–54.0)	88.0% (70.0–95.8)	92.7% (67.8–99.2)
BNT162b2, Pfizer/BioNTech	75.8% (9.7–94.7)	71.8% (28.6–89.4)	90.7% (67.7–98.3)	95.6% (89.4–98.6)
NVX-CoV2373, Novavax	NA	NA	NA	79.5% (46.8–92.1)

NA, no data available for review.

## Data Availability

Data sharing not applicable.
